# Laparoscopic liver resection versus radiofrequency ablation for hepatocellular carcinoma within Milan criteria: a meta-analysis and systematic review

**DOI:** 10.3389/fonc.2024.1442499

**Published:** 2024-11-19

**Authors:** Lin Xu, Zhenyu Lin, Dong Chen, Zhangkan Huang, Xiaozhun Huang, Xu Che

**Affiliations:** Department of Hepatobiliary Surgery, National Cancer Center/National Clinical Research Center for Cancer/Cancer Hospital & Shenzhen Hospital, Chinese Academy of Medical Sciences and Peking Union Medical College, Shenzhen, China

**Keywords:** hepatocellular carcinoma, hepatectomy, radiofrequency ablation, Milan criteria, meta-analysis

## Abstract

**Background:**

Minimally invasive techniques have significantly gained popularity for hepatocellular carcinoma (HCC) based on the Milan criteria. However, whether or not laparoscopic liver resection (LLR) or radiofrequency ablation (RFA) is a better treatment option remains debatable. We conducted a meta-analysis to review the published data comparing LLR and RFA for HCC through Milan criteria depending on tumor recurrence risk and survival.

**Methods:**

PubMed, OvidSP, Web of Science, and Cochrane Library databases were searched from inception to December 31, 2023. The studies comparing the outcomes and methods between LLR and RFA for HCC within the Milan criteria were included.

**Results:**

We recruited 19 cohort studies with 2532 patients. The postoperative complication rate was low, and hospital stays were shorter in the RFA group than in the LLR group. The total tumor recurrence, the local tumor recurrence rate, and the intrahepatic tumor recurrence rate were lower within the LLR group than in the RFA group. There was no significant difference in the extrahepatic recurrence rate between the two groups. Moreover, no significant differences were observed between the groups concerning 1-, 3-, and 5-year overall survival (OS) and 1-year recurrence-free survival (RFS). However, 3-year and 5-year RFS were better within the LLR group than among the RFA group.

**Conclusions:**

The treatment of HCC within the Milan criteria is moving toward multidisciplinary and minimally invasive approaches. Our meta-analysis identified a lower postoperative complication rate and higher recurrence rate for RFA than LLR. RFA could be an alternative treatment due to its comparable long-term efficacy with LLR.

## 1 Introduction

Hepatocellular carcinoma (HCC) is one of the most common global malignancies and causes nearly 782,000 deaths annually (1). Hepatic resection offers the therapeutic possibility of eradicating satellite tumor lesions and microscopic tumor emboli in adjacent vasculature. However, it is associated with the destruction of non-tumor liver parenchyma (2). Laparoscopic liver resection (LLR) has superior short-term, including shorter operative time, decreased blood loss, shorter hospital stays, decreased overall morbidity, and similar long-term efficacy compared with open surgery (3), even among the selected cirrhotic patients (4). Radiofrequency ablation (RFA) is an efficient local hyperthermic ablative therapy inducing homogeneous necrosis of the target tumor and providing an adequate margin of non-tumorous tissue. Thus, the American Association for the Study of Liver Disease (AASLD) (5) clinical practice guideline recommends surgical resection and percutaneous ablation therapy for patients having early HCC.

Although LLR and RFA offer favorable short- and long-term outcomes for treating HCC, there is limited evidence to indicate which procedure is more suitable for early-stage HCC. Previously published meta-analysis (6) depicted that the RFA group had a lower complication rate. In contrast, LLR had a significantly better 1- and 3-year recurrence-free survival (RFS) and 5-year overall survival (OS) than the RFA group. However, the studies included in the meta-analysis (6) showed a different definition of the ‘small HCC.’ The most commonly used descriptions were the BCLC stage 0/A or the Milan criteria. However, the tumor size ranged from <3cm to <6.5cm. Tumor size is one of the essential factors in considering ablative treatment as the efficacy in complete ablation diminished with larger tumor size, and local tumor progression was more frequent within more extensive tumors (7). Moreover, with the improvements in surgical techniques and laparoscopic instruments, LLR is no longer limited to HCC located in the anterolateral (AL). The advantages and safety of LLR for HCC found in the posterosuperior (PS) segments of the liver have been widely accepted (8, 9). Thus, whether LLR or RFA is a better treatment option among early-stage HCC remains debatable without a globally accepted treatment algorithm.

We performed an updated meta-analysis to review published literature comparing LLR and RFA for HCC within the Milan criteria to minimize the potential selection bias.

## 2 Materials and methods

This meta-analysis followed the criteria defined by the Preferred Reporting Items for Systematic reviews and Meta-Analyses statements (10).

## 3 Data sources and search strategy

A literature search was performed up to December 31, 2023, without region or publication type restriction for only English studies. Primary sources were PubMed, OvidSP, Web of Science, and Cochrane Library databases. In combination, medical subject headings (MeSH) and free-text words were utilized to explore randomized controlled trials (RCTs) and observational studies. The following MeSH terms and their combinations were examined in the title/abstract: “liver neoplasms,” “laparoscopic,” and “radiofrequency or ablation” (The search strategy in **Supplementary Materials**). Titles and abstracts were reviewed, and the candidate articles were identified. Additionally, the related article function was incorporated to broaden the search. Moreover, the computer search was supplemented using a manual search of the reference lists of all the identified studies, review articles, and conference abstracts. Two independent researchers were involved in the search (Lin Xu and Chunling Wang). Differences were resolved through consensus, and disagreements were resolved by adjudicating with senior authors (Xinyu Bi and Xiaozhun Huang).

## 4 Inclusion and exclusion criteria

Inclusion criteria for studies were: (1) all the patients diagnosed with HCC based on the cytohistological evidence from liver biopsy specimens or based on the diagnostic criteria for HCC used by the AASLD during the absence of biopsy evidence; (2) comparisons of the outcomes and methods between LLR and RFA for HCC within the Milan criteria; (3) Milan criteria is defined as the maximum tumor diameter of less than 3 cm and several intrahepatic tumors no greater than 3, or a single intrahepatic lesion having a diameter of less than 5 cm.

Exclusion criteria for studies were: (1) relevant data could not be extracted or determined; (2) non-human experimental study; (3) editorial, letter to the editor, review article, case report, or another such publication type.

## 5 Data extraction and outcomes of interest

After removing the duplicate studies, titles and abstracts of the search items were screened and sequentially excluded based on the eligibility criteria (Lin Xu and Xiaozhun Huang). If uncertainty remained concerning the title and abstract, then the two investigators independently assessed the full text (Xiaozhun Huang and Xu Che). Any discrepancies were resolved by consensus after a discussion. Primary outcomes were perioperative (operative time, complete resection/necrosis, morbidity, and hospital stay length), tumor recurrence rate, and survival outcomes. The Clavien–Dindo grading system was utilized to classify postoperative complications (11).

## 6 Quality assessment and statistical analysis

The completeness, plausibility, and integrity of the incorporated data were reviewed before being included in a single database. The methodological quality of retrospective studies was assessed through the modified Newcastle–Ottawa scale (mNOS) (12, 13), which had three factors: patient selection, comparability of study groups, and outcome assessment. Each study was provided stars based on a score of 0–9, with studies receiving eight or more stars depending on high quality. Any discrepancies were resolved through consensus. The meta-analysis was performed with the Review Manager 5.3 software (Cochrane Collaboration, Oxford, UK). The weighted mean difference (WMD) and odds ratio (OR) compared the continuous and dichotomous variables. All the results were reported using 95% confidence intervals (CI). Statistical heterogeneity among the included studies was assessed through the chi-square test. Thus, a P-value of <0.05 was considered significant, and heterogeneity was quantified through the *I* ^2^ statistic. In the event of significant heterogeneity among the included studies, the random-effects model was utilized for pooled analyses; otherwise, the fixed-effects model was incorporated (14). Publication bias was examined through the Stata 12.0 software (Stata Corporation, College Station, TX, USA).

## 7 Results

### 7.1 Search results

**Figure 1:**
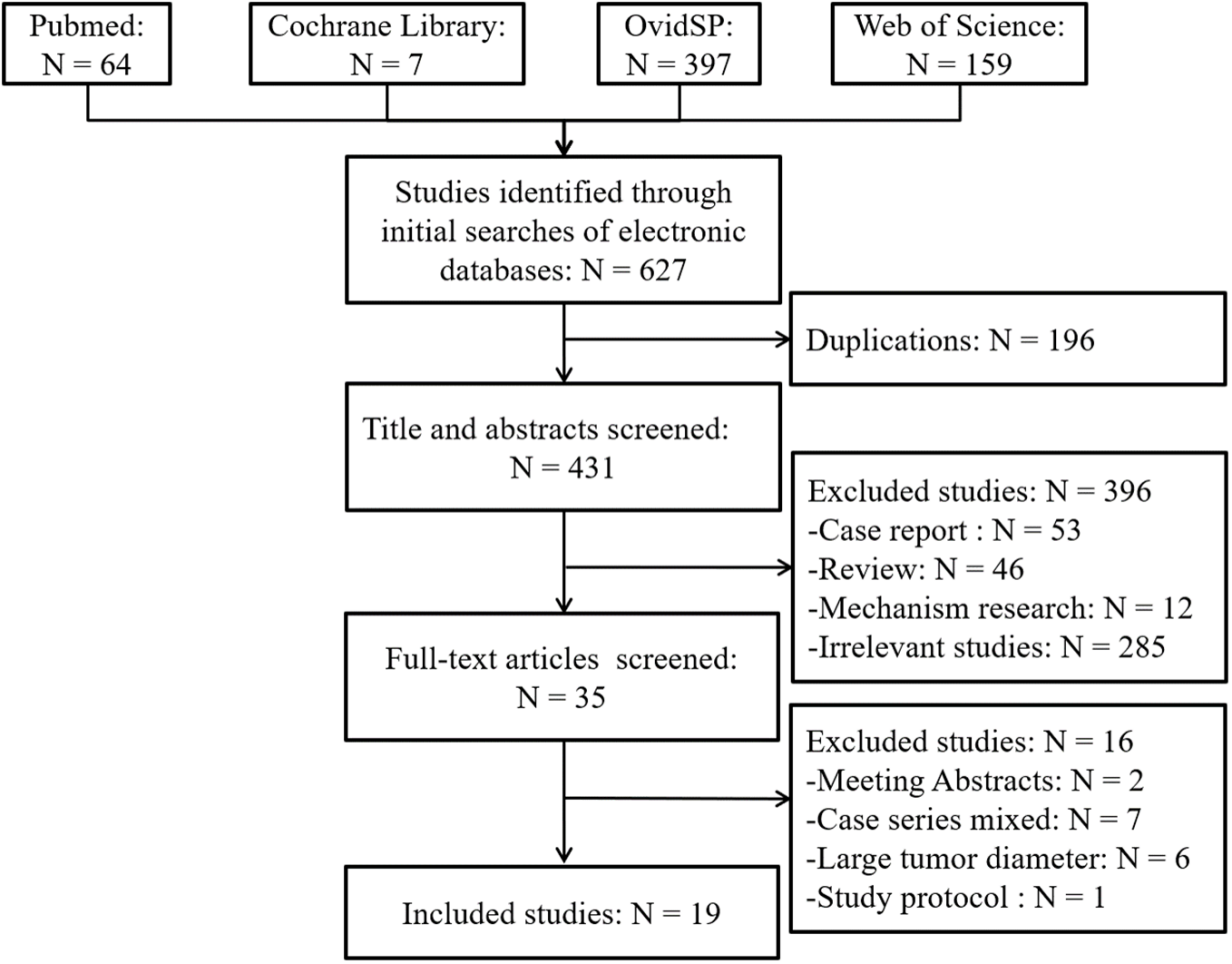
Flow diagram of the identified, included, and excluded studies.

A schematic illustration of literature search and study selection criteria is depicted in **Figure 1**. A total of 627 articles were identified through the initial search of the biomedical databases. Of these, 196 were excluded because of duplication, and 396 were excluded after reviewing the titles and/or abstracts since they were considered irrelevant. Thus, the full texts of 35 articles were reviewed. Sixteen studies were excluded, two were available only as abstracts, five involved case series that included a multimodal open and laparoscopic approach, one was study protocol, and 2 included patients with liver metastases. The population of the other six studies did not match the Milan criteria. Moreover, the remaining 19 studies (8, 9, 15–31) compared LLR and RFA in patients with early-stage HCC based on the Milan criteria. No additional studies were identified based on the manual screening of reference lists of these studies and the review articles. The agreement between the two reviewers was 100% for study selection and quality assessment of the trials.

### 7.2 Characteristics of the included studies

The characteristics of the 19 studies included in the meta-analysis have been summarized in **Table 1**. They were genuinely representative studies published between 2015 and 2023. The sample size ranged between 40 and 354, having a total of 2532 participants (1130 within the LLR group [44.6%] and 1402 within the RFA group [55.4%]). The tumor size among the RFA group was smaller than in the LLR group (P = 0.0001**; Supplementary Figure 1A**). The rate of Child-Pugh A was much higher in RFA than within the LLR group (P = 0.02; **Supplementary Figure 1B**). The rate of cirrhosis was indifferent (P = 0.33; **Supplementary Figure 1C**), and no significant difference in the rate of single tumor number between the two groups (P = 0.14; **Supplementary Figure 1D**).

**Table 1:** Summary of characteristics of included studies.

Study	Location/year	Number of patients		Number of nodules		Sex Ratio (M: F)		Age		Childs-Pugh (A: B)		Tumor size (cm)		HBV: HCV		Tumor number (solitary: multiple)	
		LLR	RFA	LLR	RFA	LLR	RFA	LLR	RFA	LLR	RFA	LLR	RFA	LLR	RFA	LLR	RFA
Cheng KC	China/2022	99	31	102	34	82:17	22:9	63.60±9.86	65.48±11.73	96:2	29:2	2.31±1.93	1.14±0.70	82:12	22:8	96:3	28:3
Chong CC	China/2020	59	59	NR	NR	46:13	46:13	57.7±10.5	59.3±11.0	59:0	58:1	2.0 (1.6–2.8)	2.3 (1.5–2.7)	48:4	48:4	56:3	56:3
Conticchio M	Italy/2022	58	58	58	58	44:14	37:21	75.4 (69.5–86.5)	74 (70–87)	50:8	52:6	3 (1–3)	2 (1.2–3)	16:30	4:39	58:0	58:0
Harada N	Japan/2016	20	20	20	20	9:11	11:9	74±6	73±9	NR	NR	1.8±0.6	1.6±0.6	2:15	1:14	19:2	18:2
Ito T	Japan/2016	27	27	27	27	16:11	15:12	69 (66–72)	71 (68–74)	26:1	26:1	2.0 (1.7–2.2)	1.7 (1.5–1.9)	6:17	5:22	25:2	21:6
Kim S	Korea/2021	61	61	61	61	43:18	52:9	59.4	62.2	59:2	56:5	2.29±0.8	2.2 ±0.8	43:3	46:3	61:0	61:0
Ko SE	Korea/2021	60	29	60	29	42:18	24:5	55.8 ± 9.0	60.0 ± 9.8	NR	NR	2.1 (1.0–2.9)	1.6 (1.0–2.8)	42:4	18:2	60:0	29:0
Lai C	China/2016	28	33	30	36	24:4	29:4	56.5±12.6	62.8±11.3	28:0	28:0	3.0±1.1	2.4±0.9	23:1	25:1	26:2	30:3
Lee DH	Korea/2021	118	118	118	118	91:27	88:30	59.5±8.7	60.5±10.3	118:0	118:0	1.84±0.56	1.87±0.51	90:10	84:12	118:0	118:0
Lin CH	China/2020	36	39	36	39	27:9	25:14	>70 (8)	>70 (14)	36:0	39:0	1.7±0.25	1.5±0.22	25:9	25:17	36:0	39:0
Ogiso S	Japan/2021	85	136	100	159	62:23	98:38	69 (46–88)	73 (47–87)	73:12	110:26	2.1 (0.8–3)	1.6 (0.5–3)	20:47	21:85	73:12	115:21
Pan YX	China/2020	118	236	NR	NR	101:17	206:30	53 (45.2-61)	56 (45-64)	NR	NR	2.5(1.85-3.5)	2.55(1.9-3.23)	NR	NR	98:20	199:37
Santambrogio R	Italy/2018	59	205	59	205	42:17	152:53	68 ± 9	69 ± 9	59:0	205:0	2.09±0.67	1.91±0.58	8:43	29:136	59:0	205:0
Song J	China/2016	78	78	78	78	70:8	70:8	48 (44, 57)	48 (43, 58)	78:0	76:2	NR	NR	73:NR	77:NR	78:0	78:0
Wang LN	China/2020	70	90	NR	NR	51:19	62:28	58.34±7.98	59.23±9.24	54:16	63:27	NR	NR	NR	NR	NR	NR
Wu D	China/2020	35	20	35	20	30:5	17:3	61.8±8.51	61.6±6.67	29:6	17:3	3.56 ± 0.68	3.50 ± 0.54	28:NR	15:NR	35:0	20:0
Yamashita YI	Japan/2019	38	62	NR	NR	25:13	40:22	66.9±9.1	66.5±9.5	33:5	54:8	2.4±0.9	2.0±0.6	5:30	9:45	32:6	42:20
Vitali GC	Switzerland/2015 015	45	60	45	60	30:15	52:8	61.4 (31–84)	67.3 (47–83)	40:5	45:15	2.3 (1–3)	2.1 (2.1–3)	8:NR	7:NR	45:0	60:0
Kang M	Korea/2023	36	40	36	40	25:11	34:6	57.8±11.7	61.6±13.72	36:0	40:0	2±0.57	1.5±0.51	30:0	31:1	36:0	40:0

LLR, laparoscopic liver resection; RFA, radiofrequency ablation; NR, not reported.

### 7.3 The methodological quality of the included studies

The studies were evaluated for the sources of bias through mNOS. The quality of all the included studies was generally high (**Table 2**). Of the 19 studies, six (20, 22–25, 31) had a score of 8/9, and 8 (9, 15–18, 21, 27, 30) had a score of 9/9, four studies (8, 26, 28, 29) with a score of 7/9, and only one study (19) showed a score of 6/9. Only three studies (19, 20, 28) could not mention the follow-up duration, and all the studies reported one perioperative outcome. In contrast, only the two studies (19, 29) had not reported the survival data.

**Table 2:** Risk of bias using the modified Newcastle-Ottawa Scale.

Study	Selection				Comparability	Outcome			Score
	Representativeness of exposed cohort	Selection of non-exposed cohort	Exposure	Outcome of interest not present at start	Comparability of LLR vs RFA	Assessment of outcome	Follow-up	Adequacy of follow-up	
Cheng KC	Truly representative	Same	Surgical records	Yes	Restricted, matched	Record linkage	Yes	Complete	9⭐
Chong CC	Truly representative	Same	Surgical records	Yes	Not restricted, matched	Record linkage	Yes	Complete	8⭐
Conticchio M	Truly representative	Same	Surgical records	Yes	Not restricted, matched	Record linkage	Yes	Unclear	7⭐
Harada N	Truly representative	Same	Surgical records	Yes	Restricted, matched	Record linkage	Yes	Complete	9⭐
Ito T	Truly representative	Same	Surgical records	Yes	Not restricted, matched	Record linkage	Yes	Unclear	7⭐
Kim S	Truly representative	Same	Surgical records	Yes	Restricted, matched	Record linkage	Yes	Complete	9⭐
Ko SE	Truly representative	Same	Surgical records	Yes	Restrictions, matched	Record linkage	Yes	Complete	9⭐
Lai C	Truly representative	Same	Surgical records	Yes	Not restricted, matched	Record linkage	Yes	Unclear	7⭐
Lee DH	Truly representative	Same	Surgical records	Yes	Restrictions, matched	Record linkage	Yes	Complete	9⭐
Lin CH	Truly representative	Same	Surgical records	Yes	Not restricted matched	Record linkage	Yes	Complete	8⭐
Ogiso S	Truly representative	Same	Surgical records	Yes	Restrictions, matched	Record linkage	Yes	Complete	9⭐
Pan YX	Truly representative	Same	Surgical records	Yes	Restrictions, matched	Record linkage	Yes	Complete	9⭐
Santambrogio R	Truly representative	Same	Surgical records	Yes	Not restricted matched	Record linkage	Yes	Complete	8⭐
Song J	Truly representative	Same	Surgical records	Yes	Restrictions, matched	Record linkage	Yes	Complete	9⭐
Wang LN	Truly representative	Same	Surgical records	Yes	Restrictions, matched	Record linkage	Yes	Unclear	8⭐
Wu D	Truly representative	Same	Surgical records	Yes	Not restricted matched	Record linkage	Unclear	Unclear	6⭐
Yamashita YI	Truly representative	Same	Surgical records	Yes	Not restricted matched	Record linkage	Yes	Complete	8⭐
Vitali GC	Truly representative	Same	Surgical records	Yes	Not restricted matched	Record linkage	Yes	Unclear	7⭐
Kang M	Truly representative	Same	Surgical records	Yes	Restrictions, matched	Record linkage	Yes	Unclear	8⭐

## 8 Primary outcomes

### 8.1 Perioperative outcomes

**Figure 2:**
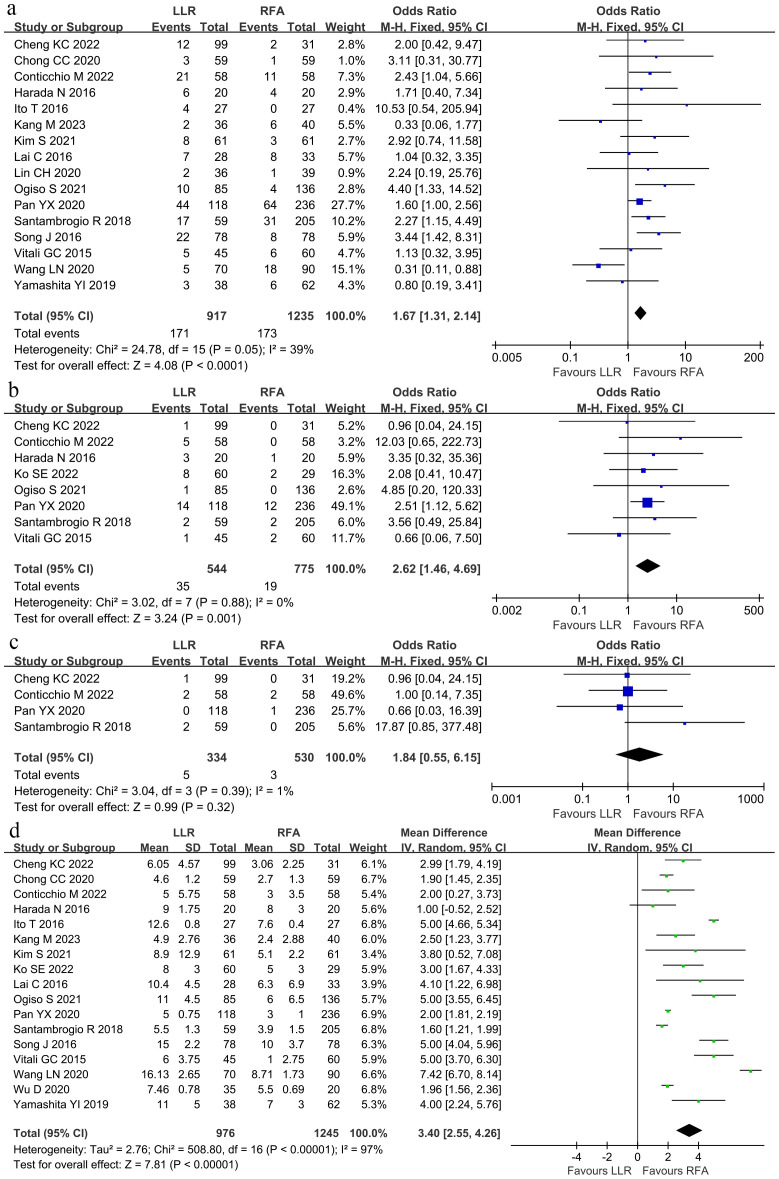
Forest plot comparing the perioperative outcomes. **(A)** Overall perioperative complication rate. **(B)** Above Grade 3 perioperative complication rate. **(C)** The in-hospital death. **(D)** Length of hospital stay.

After pooling the data from the studies based on postoperative outcomes, 16 studies (8, 9, 16, 18, 20–31) depicted the overall perioperative complication rate. The perioperative complication rate was higher among the LLR group than in the RFA group (18.6% vs. 14.0%, OR 1.67, 95%CI 1.31–2.14; P < 0.0001; **Figure 2A**). Moderate heterogeneity was noted (χ^2^ = 24.78; P = 0.05, *I^2^* = 39%) through the fixed effects model. Eight studies (8, 9, 15, 16, 21, 25, 26, 30) depicted the rate of perioperative complications above Grade 3, classified by the Clavien–Dindo grading system. The above Grade 3 perioperative complication rate was higher within the LLR group than within the RFA group (6.43% vs. 2.45%, OR 2.62, 95%CI 1.46–4.69; P = 0.001; **Figure 2B**). No heterogeneity was observed (χ^2^ = 3.02; P = 0.88, *I^2^* = 0) through the fixed effects model.

Based on the various treatment-related complications (**Table 3**), there was no variation in the internal hemorrhage rate, postoperative biliary complication, pneumonia, effusion or infection within the operative area, incision-related complications, ascites, arrhythmia, intraperitoneal complications, pulmonary complications, and organ failure. The rate of blood transfusion rate among the LLR group was significantly higher than in the RFA group (8.83% and 1.55%, respectively; OR 4.39; 95% CI 2.19–8.78; P < 0.0001). Moreover, there was no heterogeneity in the data reported from the included studies (χ^2^ = 6.47; P = 0.60, *I* ^2^ = 0).

**Table 3:** Meta-analysis results of all available studies in complication treatment related.

Postoperative outcomes	No. Cohorts	No. Patients		Heterogeneity test		Model	OR	95%CI	*P*
		LLR	RFA	*I* ^2^	*P*				
Internal hemorrhage	7	374	383	0	0.70	Fixed	1.77	0.71-4.38	0.22
Biliary complication	7	379	497	18	0.29	Fixed	1.33	0.61-2.87	0.47
Pneumonia	4	305	257	0	0.77	Fixed	1.73	0.62-4.82	0.30
Organ failure	3	137	283	0	0.67	Fixed	4.00	0.93-17.13	0.06
Effusion or infection in the operative area	5	260	179	0	0.58	Fixed	2.47	0.71-8.56	0.16
Incision-related complications	8	391	565	0	0.64	Fixed	1.71	0.84-3.47	0.14
Ascites	8	490	752	69	0.002	Random	0.02	-0.02-0.06	0.30
Arrhythmia	3	275	325	0	0.73	Fixed	2.29	0.44-11.94	0.33
Intraperitoneal complications	3	199	317	0	0.39	Fixed	2.50	1.12-5.59	0.03
Pulmonary complications	6	397	534	0	0.60	Fixed	1.37	0.46-4.11	0.57
Rate of blood transfusion	9	487	517	0	0.60	Fixed	4.39	2.19-8.78	<0.0001

LLR, laparoscopic liver resection; RFA, radiofrequency ablation; OR, odds ratio; 95%CI, 95% confidence intervals.

Four studies (8, 9, 21, 25) described the in-hospital death, a meta-analysis with the fixed effects model described no significant difference in mortality between the two groups (1.49% and 0.57%, respectively; OR 1.84; 95%CI 0.55–6.15; P = 0.32), and no statistical heterogeneity (χ^2^ = 3.04; P = 0.39, *I* ^2^ = 1%; **Figure 2C**). Thus, the length of the hospital stay was longer (WMD, 3.40 days shorter within the RFA group; 95% CI 2.55–4.26; P < 0.00001; **Figure 2D**) within the LLR group than in the RFA group. Significant heterogeneity was observed (τ^2^ = 2.76, χ^2^ = 508.8; P < 0.00001, *I^2^* = 97%) with the random effects model.

**Figure 3:**
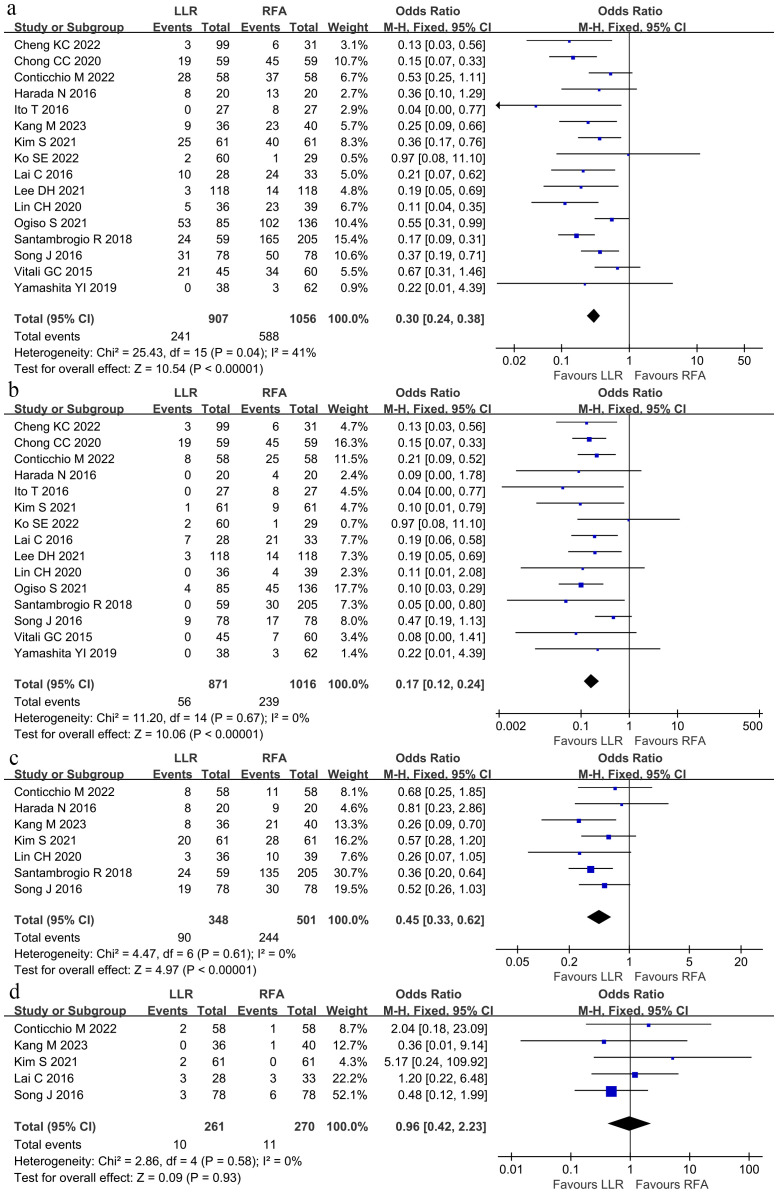
Forest plot for tumor recurrence. **(A)** The total tumor recurrence was lower within the LLR group than in the RFA group. **(B)** The local tumor recurrence rate was lower in the LLR group than in the RFA group. **(C)** The intrahepatic tumor recurrence rate was lower within the LLR group than in the RFA group. **(D)** There was no significant difference in the extrahepatic recurrence rate between the LLR group and the RFA group.

The total tumor recurrence rate was defined as intrahepatic and metastasis rates. Sixteen studies (8, 9, 15–18, 22–31) reported the total tumor recurrence was lower in the LLR group than in the RFA group (26.57% vs. 55.68%; OR 0.30; 95% CI 0.24–0.38; P < 0.00001), and moderate heterogeneity was observed (χ^2^ = 25.43; P = 0.04, *I^2^* = 41%; **Figure 3A**). Among the 15 studies, the local tumor recurrence rate was lower in the LLR group than within the RFA group (6.43% vs. 23.52%; OR 0.17; 95% CI 0.12–0.24; P < 0.00001), and no heterogeneity was observed (χ^2^ = 11.20; P = 0.67, *I^2^* = 0; **Figure 3B**). Similarly, the intrahepatic tumor recurrence rate was lower within the LLR group than within the RFA group (25.86% vs. 48.70%; OR 0.45; 95% CI 0.33–0.62; P < 0.00001), and no heterogeneity was observed (χ^2^ = 4.47; P = 0.61, *I^2^* = 0; **Figure 3C**). There was no significant difference inside the extrahepatic recurrence rate between the two groups (3.83% vs. 4.07%; OR 0.96; 95% CI 0.42–2.23; P = 0.93), and no heterogeneity was observed (χ^2^ = 2.86; P = 0.58, *I^2^* = 0; **Figure 3D**).

### 8.2 Survival outcomes

**Figure 4:**
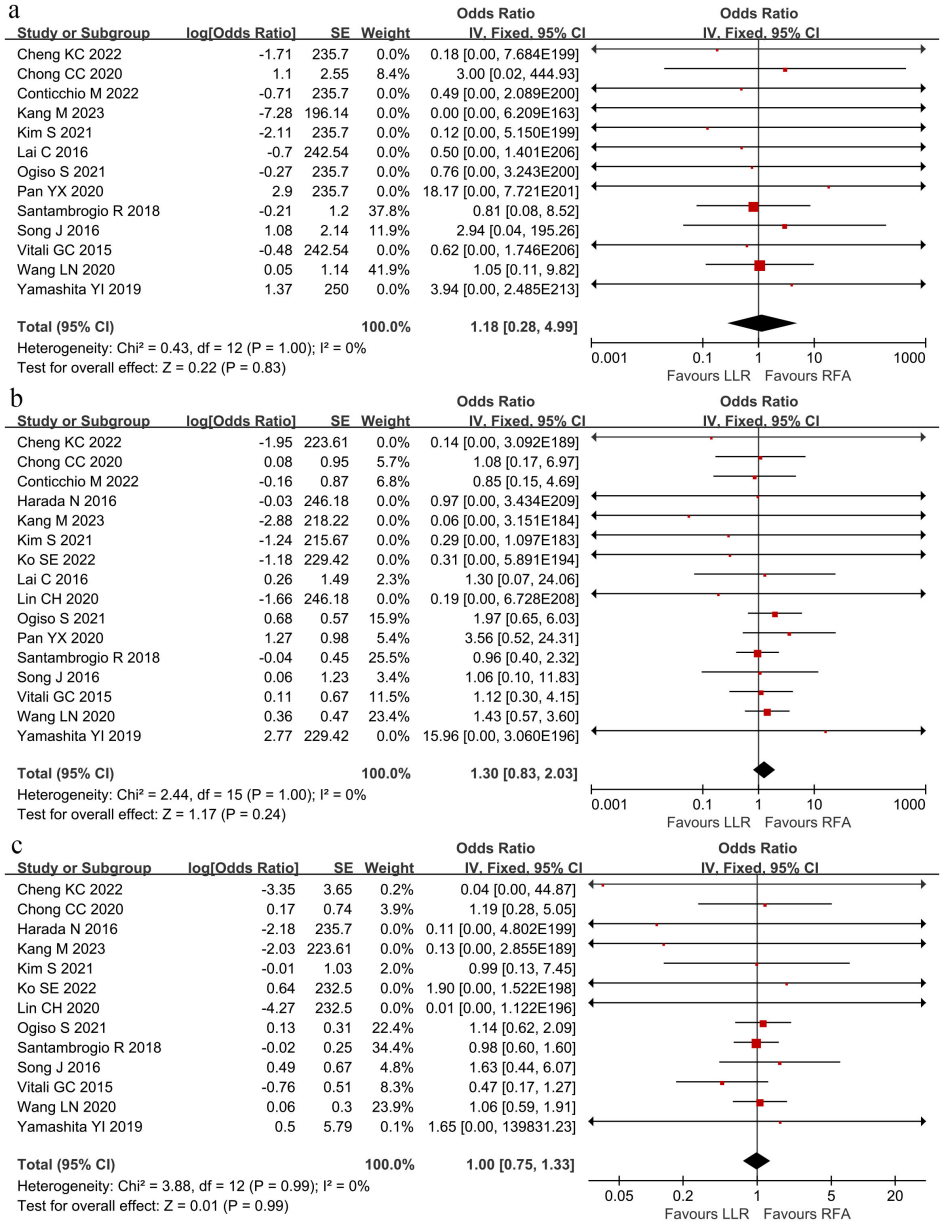
The pooled results depicted no significant differences in overall survival at **(A)** 1-year overall survival, **(B)** 3-year overall survival, or **(C)** 5-year overall survival between LLR and RFA. Hazard ratio at 1-, 3- and 5-years were 1.18 (95% CI 0.28–4.99), 1.30 (95% CI 0.83–2.03), and 1.00 (95% CI 0.75–1.33).

The pooled analysis compared OS among the groups at 1, 3, and 5 years through the hazard ratio (HR). The meta-analysis depicted no significant differences in OS at 1, 3, and 5 years (**Figures 4A–C**). The HR at 1, 3, and 5 years were 1.18 (95% CI 0.28–4.99), 1.30 (95% CI 0.83–2.03), and 1.00 (95% CI 0.75–1.33), respectively. No heterogeneity was observed in the data for the 1-, 3-, and 5-year time points.

**Figure 5:**
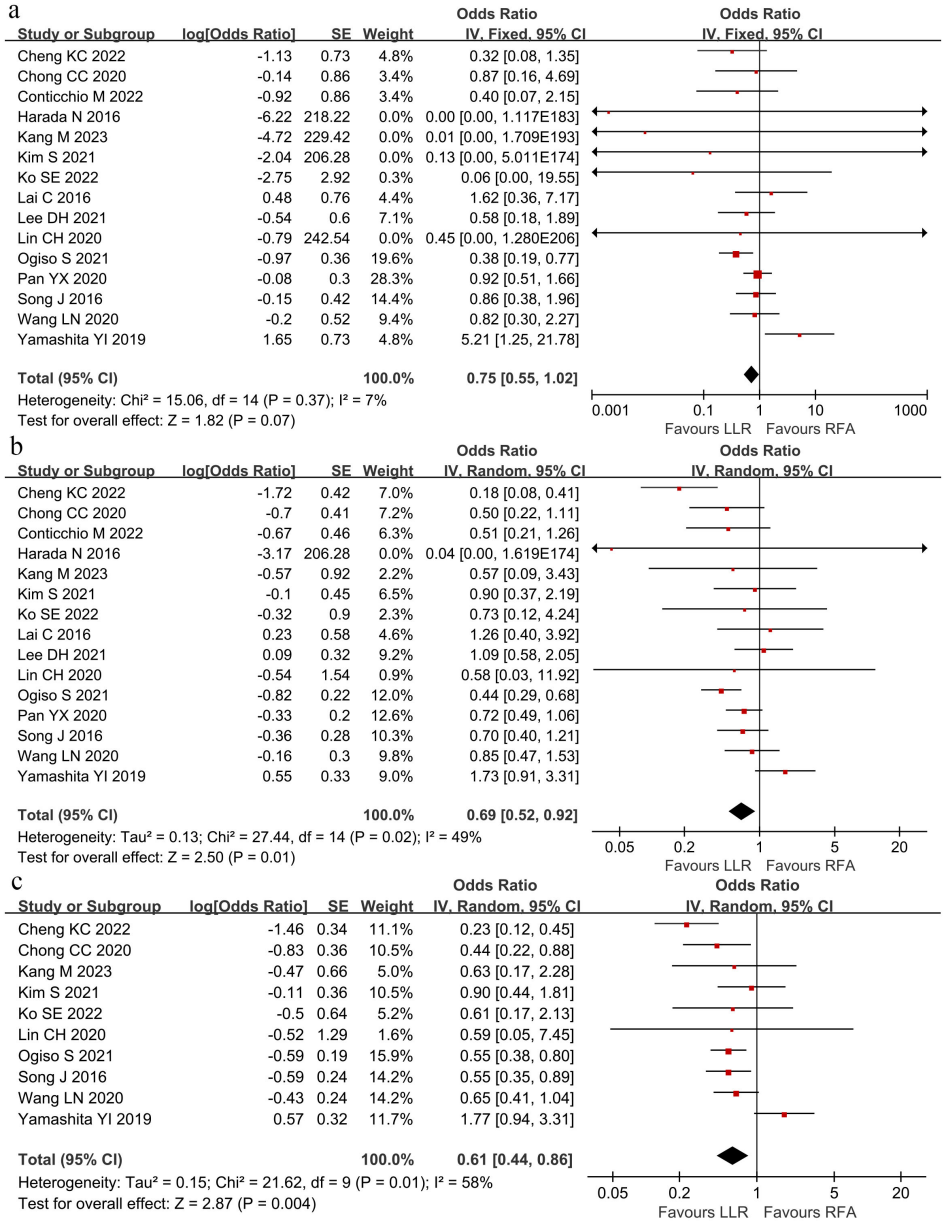
The pooled results revealed no significant differences in recurrence-free survival at **(A)** 1-year recurrence-free survival, but the LLR group had better **(B)** 3-year recurrence-free survival and **(C)** 5-year recurrence-free survival than the RFA group. Hazard ratio at 1-, 3- and 5-years were 0.75 (95% CI 0.55–1.02), 0.69 (95% CI 0.52–0.92) and 0.61 (95% CI 0.44–0.86).

RFS was compared between the groups at 1 and 3, and 5 years. Overall, 1-year RFS did not significantly differ between the groups (HR 0.75; 95% CI 0.55–1.02; P = 0.07; **Figure 5A**). In contrast, RFS at 3- and 5-years was better within the LLR group than in the RFA group (3-year HR 0.69; 95% CI 0.52–0.92; P = 0.01; **Figure 5B**; 5-year HR 0.61; 95% CI 0.44–0.86; P = 0.004; **Figure 5C**). The *I^2^* was 7%, 49%, and 58% for 1-, 3-, and 5-year RFS. Sensitivity analysis revealed that no study could significantly affect the pooled HR.

### 8.3 Subgroup analysis

We performed subgroup analyses of the different characteristics of included studies (**Table 4**). There were six subgroups containing the tumor single in the included studies. Using Propensity Score Matching (PSM), the largest tumor diameter was ≤ 3 cm, the population in studies was from Asia, the method in RFA was the percutaneous approach, and high-quality studies were defined as mNOS score ≥ 7. In the single tumor subgroup, the 3-year RFS did not differ significantly between the groups (HR 0.92; 95% CI 0.69–1.24). In the PSM subgroup studies, there was no significant difference in the extrahepatic recurrence rate between the two groups (OR 0.61; 95% CI 0.34–1.10). Not only was the 3- and 5-year RFS lower in the RFA group, but the percutaneous RFA subgroup showed the 1-year RFS also lower than the LLR group (HR 0.69; 95% CI 0.49–0.96).

**Table 4:** Meta-analysis results of subgroup analysis.

Subgroup analysis	Single nodular	PSM cohort	Largest tumor diameter ≤ 3 cm	Asia group	LLR vs PRFA	High quality studies (≥ 7 scores)
Total complication (OR [95%CI])	2.10 [1.43, 3.07]	1.92 [1.32, 2.80]	2.17 [1.46, 3.23]	1.71 [1.06, 2.77]	1.78 [1.01, 3.13]	1.78 [1.24, 2.54]
Major complication (OR [95%CI])	2.72 [1.02, 7.23]	2.71 [1.38, 5.34]	2.92 [1.23, 6.97]	2.46 [1.27, 4.78]	2.46 [1.27, 4.78]	2.62 [1.46, 4.69]
Total tumor recurrence (OR [95%CI])	0.32 [0.24, 0.42]	0.27 [0.19, 0.40]	0.32 [0.24, 0.43]	0.29 [0.22, 0.38]	0.34 [0.25, 0.47]	0.31 [0.24, 0.39]
Local tumor recurrence (OR [95%CI])	0.31 [0.24, 0.39]	0.16 [0.10, 0.26]	0.14 [0.09, 0.21]	0.17 [0.12, 0.26]	0.18 [0.12, 0.29]	0.17 [0.12, 0.24]
Intrahepatic tumor recurrence (OR [95%CI])	0.45 [0.32, 0.63]	0.61 [0.34, 1.10]	0.44 [0.28, 0.68]	0.52 [0.34, 0.81]	0.57 [0.36, 0.91]	0.48 [0.34, 0.67]
1-year OS (HR [95%CI])	1.10 [0.14, 8.58]	3.00 [0.02, 444.09]	1.03 [0.12, 8.63]	1.47 [0.24, 9.23]	1.32 [0.18, 9.48]	1.18 [0.28, 5.00]
3-year OS (HR [95%CI])	0.99 [0.52, 1.89]	1.41 [0.49, 4.05]	1.17 [0.68, 2.03]	1.63 [0.90, 2.95]	1.70 [0.91, 3.19]	1.30 [0.83, 2.03]
5-year OS (HR [95%CI])	0.91 [0.61, 1.37]	1.02 [0.32, 3.25]	0.95 [0.67, 1.35]	1.13 [0.77, 1.65]	1.12 [0.76, 1.66]	1.00 [0.75, 1.33]
1-year RFS (HR [95%CI])	0.67 [0.36, 1.26]	0.71 [0.45, 1.12]	0.60 [0.37, 1.00]	0.76 [0.56, 1.05]	0.69 [0.49, 0.96]	0.75 [0.55, 1.02]
3-year RFS (HR [95%CI])	0.92 [0.69, 1.24]	0.64 [0.50, 0.83]	0.69 [0.53, 0.91]	0.69 [0.58, 0.84]	0.65 [0.53, 0.79]	0.69 [0.57, 0.82]
5-year RFS (HR [95%CI])	0.50 [0.35, 0.72]	0.45 [0.31, 0.66]	0.68 [0.51, 0.90]	0.61 [0.50, 0.74]	0.55 [0.44, 0.68]	0.61 [0.50, 0.74]

PSM, Propensity Score Matching; LLR, laparoscopic liver resection; PRFA, percutaneous radiofrequency ablation; OR, odds ratio; 95%CI, 95% confidence intervals; HR, hazard ratio; OS, overall survival; RFS, recurrence-free survival.

### 8.4 Publication bias

**Figure 6:**
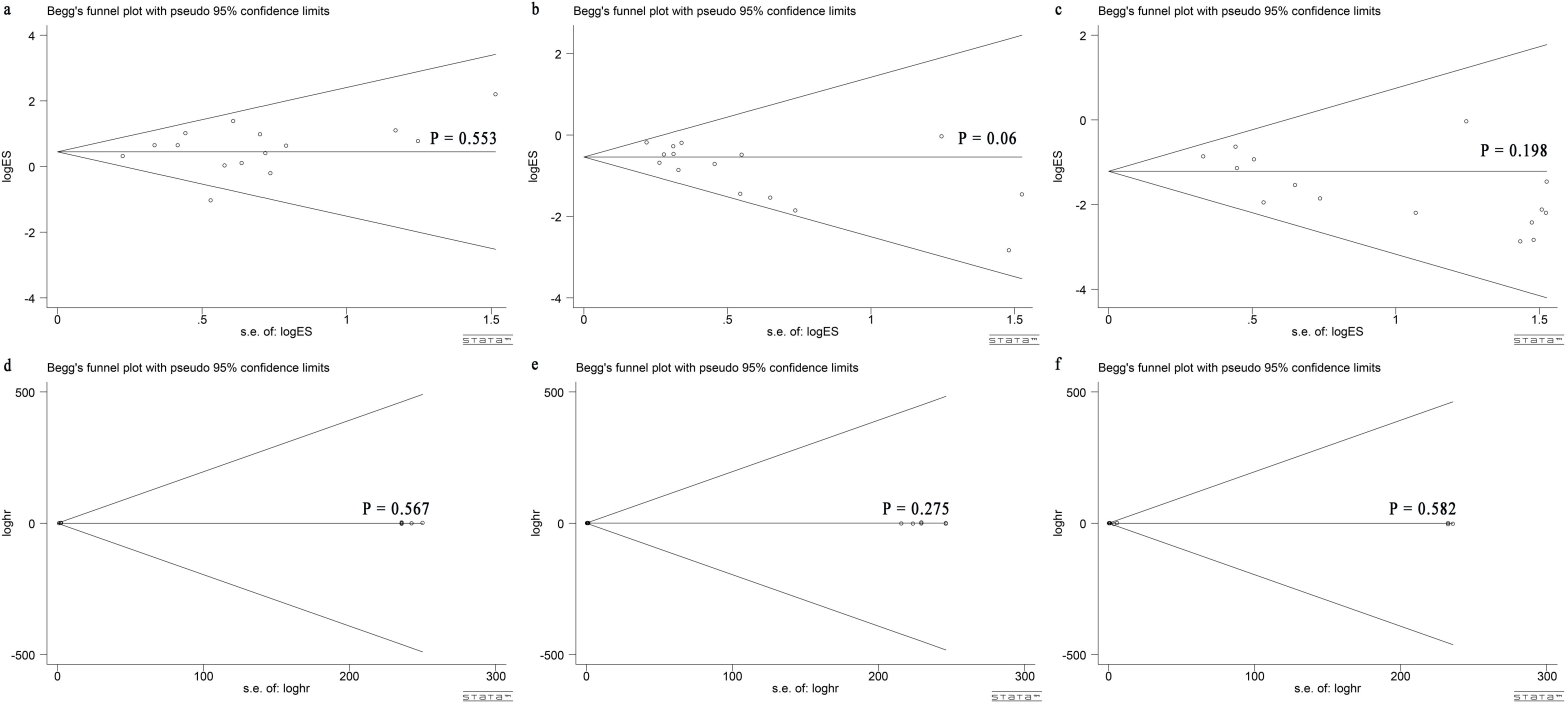
Begg’s test for comparing LLR and RFA for HCC within Milan criteria showed no publication bias. Begg’s funnel plot with pseudo 95% CI limits in **(A)** perioperative complication rate (P = 0.553), **(B)** total tumor recurrence rate (P = 0.06), **(C)** local recurrence rate (P = 0.198), **(D)** 1-OS (P = 0.567), **(E)** 3-OS (P = 0.275) and **(F)** 5-OS (P = 0.582).

Based on the Begg’s rank correlation test, no significant difference was observed in publication bias among perioperative complication rate (P = 0.553; **Figure 6A**), the total tumor recurrence rate (P = 0.06; **Figure 6B**), the local recurrence rate (P = 0.198; **Figure 6C**), and 1-OS (P = 0.567; **Figure 6D**), 3-OS (P = 0.275; **Figure 6E**), 5-OS (P = 0.582; **Figure 6F**), respectively.

## 9 Discussion

The current meta-analysis of 18 cohort studies compared perioperative and oncological outcomes between LLR and RFA for HCC within the Milan criteria. The main findings obtained from our meta-analysis showed that RFA had a lower postoperative complication rate and similar oncological outcomes. However, a higher recurrence rate and RFS compared with LLR. The previous meta-analysis indicated that the laparoscopic approach is related to improved short-term outcomes concerning wider resection margins, reduced intraoperative blood loss (32), reduced need for transfusions, postoperative bile leakage (33), postoperative infection (32), and reduced morbidity rates and shorter lengths of hospital stay (33), than with open hepatectomy (34–36). Several randomized controlled trials and meta-analyses have compared the efficacy of RFA and open hepatectomy. RFA was an effective treatment during early-stage HCC, having a comparable prognostic outcome and a lower complication rate than open hepatectomy (37, 38). Therefore, RFA and LLR have been recommended to treat HCC within the Milan criteria. Hence, several studies (24, 27, 39) focused on comparing these two minimally invasive methods for the curative treatment of HCC between the LLR and RFA with conflicting conclusions.

Overall morbidity is crucial in our review to assess the safety of the method. Our meta-analysis showed the postoperative complication rate of LLR was 19.2%, and the major perioperative complication (above Grade 3 classified by the Clavien–Dindo grading system) was 6.43% in the LLR group, which was similar to previously reported (36). However, pooled analysis established the benefits of RFA with moderate heterogeneity and its demonstrable effect among different settings. Among the various subgroup analyses of treatment-related complications, there was no difference in intra-abdominal, extraperitoneal, and systemic complications. Such discrepancies could be explained to some extent by hospital volume or surgeon experience, as discussed further within the limitations of our review. However, it seems that the definitions and reporting of specific complications have not been standardized, resulting in significant discrepancies between the included studies and eventual bias.

Incomplete necrosis could cause cancer stemness or epithelial-mesenchymal transition of HCC cells and affect intrahepatic dissemination or distant metastasis (40), associated with higher local and total tumor recurrence rates with RFA. A higher local recurrence rate for a larger HCC size was due to several factors. A large number of precisely calculated overlapping coagulations was necessary for large tumors. The statistical data showed that 14 overlapping coagulations are needed to cover a 3 cm tumor and its safety margin having an electrode that produces perfect spherical coagulation of 3 cm (41). When more than one treatment session is required to achieve complete ablation, there is a risk of local recurrence (42). Larger tumors have irregular borders more frequently than small tumors, creating difficulty achieving an oncologic safety margin. Tumor size is the only independent risk factor of early recurrence after the RFA of HCC. On the other hand, based on the hepatectomy principle, the minimum required length of safety margin is 5.5 mm and 6 mm to achieve 99% and 100% micro-metastasis clearance surrounding the liver of HCC patients (43). In our meta-analysis, the tumor size in the RFA group was smaller than in the LLR group. However, the RFA group had a higher recurrent rate than the LLR group, indicating that surgical resection could be due to the removal of small tumor thrombus in the vein adjacent to the liver other than the primary tumor.

The PSM method can achieve a “quasi-randomization” effect within non-randomized controlled studies that cannot be randomized in the study design stage. Therefore, our comprehensive meta-analysis pooled high-quality studies to obtain a more systematic and robust power of the results assessing the superiority of LLR or RFA in HCC within Milan criteria. Thus, our meta-analysis conducted a subgroup that included the PSM studies, finding the pattern of tumor recurrence with all patient subsets. The total tumor recurrence rate and local recurrence rate were higher in the RFA group in the PSM subgroup. However, there were no significant differences in the intrahepatic and extrahepatic recurrence rates between the two groups. The major baseline characteristics of the subgroup were well balanced, which might reveal that incomplete necrosis was the real reason for local recurrence, and the puncture of the ablation needle did not cause intrahepatic dissemination or distant metastasis.

Previous studies have indicated that tumor control is better with laparoscopic and open RFA than with percutaneous RFA (44–46). The surgical approach provides ablation through multiple needle electrode punctures at different angles, preventing dead ends inside tumors. The laparoscopic approach is characterized by further advantages over the percutaneous approach for HCC as it allows intraoperative ultrasound examination to diagnose the otherwise undetected nodules. Thus, it provides better tumor visualization and more accurate placement of the ablation probe (47).

It has been debated whether resection or RFA could be a better treatment for early-stage HCC. Two RCTs reported that RFA is related to similar survival rates as hepatic resection (48, 49). In contrast, two other trials said that RFA is inferior to resection in patient survival and tumor recurrence (50, 51). A meta-analysis (6) in all HCC patients revealed that LLR was superior to RFA regarding the 5-year OS rate. The subgroup analysis of the small HCC confirmed an improved 5-year OS rate for the LLR group than for the RFA group. Moreover, no significant difference in the 1- and 3-year OS rates were detected.

Our meta-analysis found no significant differences between LLR and RFA concerning the long-term outcomes, including 1-, 3-, and 5-year OS and 1-year RFS. However, 3-year and 5-year RFS were better with LLR than with RFA. LLR was not superior to RFA in terms of OS. The similar OS rates in both groups can be attributed to reasonably aggressive approaches to detecting and treating tumor recurrence. Most patients had an intrahepatic recurrence, treated with resection, RFA, transarterial chemoembolization (TACE), or through salvage liver transplantation.

Independent risk factors influencing postoperative survival involve the preservation of liver function (ICG-15) and the presence of multiple tumors in different liver segments. The leading cause of death in HCC patients is tumor recurrence and gradual liver function deterioration (49). In multivariate analysis, the treatment arm (resection or RFA) was not identified as a prognostic factor for either OS or disease-free survival. Patients with a small solitary HCC and preserved liver function can be effectively treated with resection or RFA to ensure a favorable survival outcome, and adjuvant therapy could have a role after treatment (52). The trend in liver cancer treatment is toward multidisciplinary and minimally invasive methods, and various patients require treatment having different treatment modalities.

The choice of surgical approach for HCC patients remains challenging for surgeons, radiologists, and hepatologists. Invasive procedures, including LLR, remain widely accepted due to their proven influence on prognosis (53, 54). In contrast, evidence is developing for all types of RFA approaches, which are well-tolerated but correlated with high recurrence rates (55).

There are some limitations to this study. First, all included studies were observational, and there was a lack of randomized data due to selection bias. Second, variations in the studied population, disease stage, surgical or ablation technique/device, and the follow-up protocol could have influenced patient outcomes. Third, the cohort sample size was relatively small, reducing the quality of the conclusions. Three studies (27–29) reported RFA was performed percutaneously, and the other two (25, 39) said RFA was performed laparoscopically. Furthermore, in the study by Santambrogio et al. (25), 22% of patients underwent microwave ablation. This approach has been reported to show similar perioperative and oncological outcomes as RFA for small HCC (56). This is the updated meta-analysis to compare different minimally invasive approaches (LLR versus RFA) for HCC with the Milan criteria. We identified several factors that could influence OS, and well-designed RCTs are necessary to compare the efficacies of the assessed treatments within specific situations.

## 10 Conclusions

Our meta-analysis observed that the postoperative complication rate is low, and the recurrence rate is higher with RFA than with LLR. RFA could be an alternative treatment because of its comparable long-term efficacy with LLR.

## Data Availability

The original contributions presented in the study are included in the article/**Supplementary Material**. Further inquiries can be directed to the corresponding authors.
