# Supplementary figures and images for: Laparoscopic liver resection versus radiofrequency ablation for hepatocellular carcinoma within Milan criteria: a meta-analysis and systematic review
Source: Front Oncol. 2024 Nov 19;14:1442499. doi: 10.3389/fonc.2024.1442499 (PMC11611894; doi:10.3389/fonc.2024.1442499)

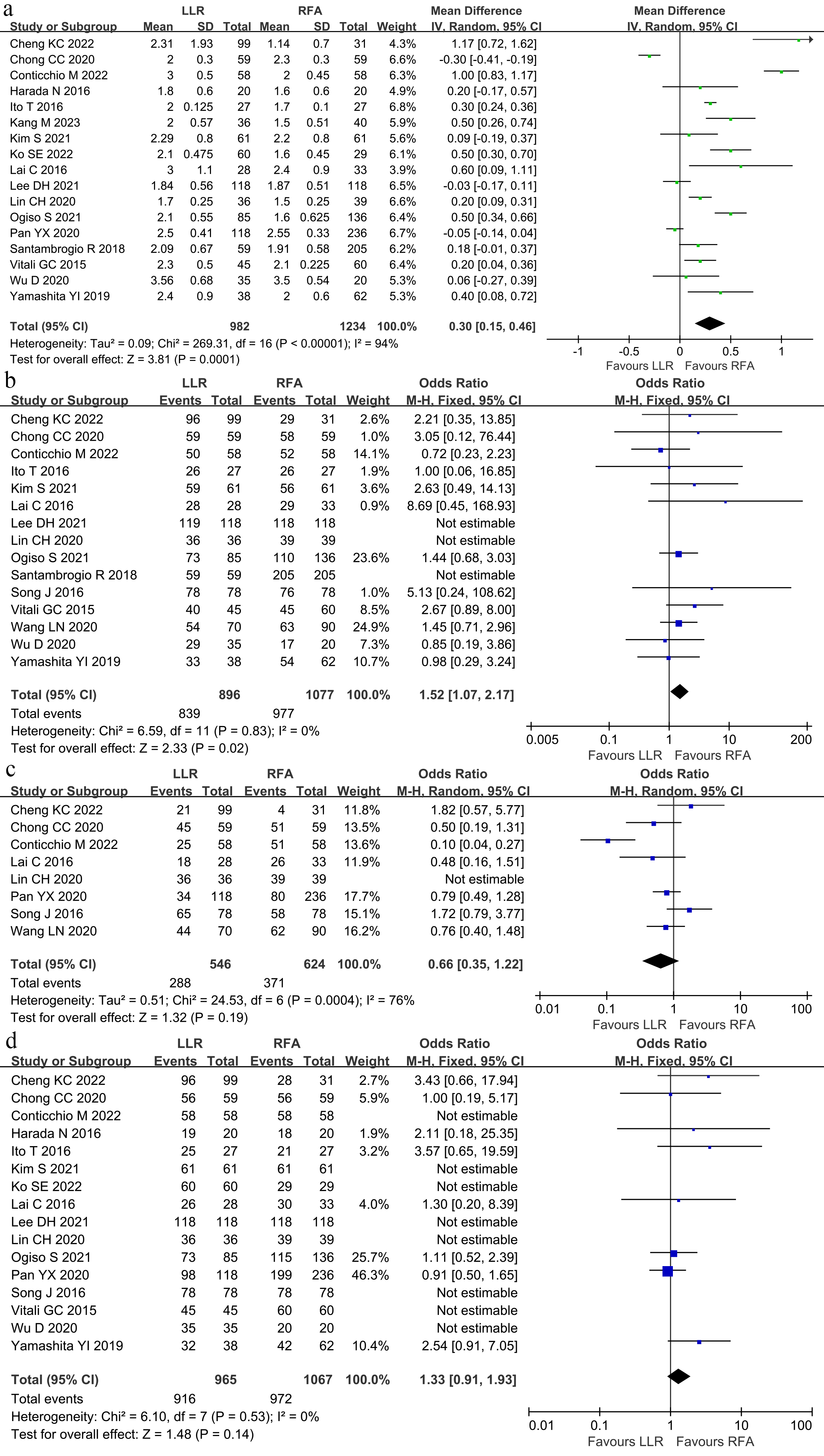

Supplement: Supplementary file 1 [file Image1.tif]
